# A New Dental College

**Published:** 1882-01

**Authors:** 


### EDITORIAL.

### A NEW DENTAL COLLEGE.

At a recent meeting of the Board of Regents of the University of California, a committee designated and appointed for the purpose, submitted the following plan of organization:

First---That a Dental Department be established in connection with the Medical Department of the University, and that the same be organized by the appointment of the following professorships: viz., Principles and Practice of Operative Dentistry; Dental Art and Mechanism; Dental Pathology and Therapeutics; Physiology; Chemistry; Anatomy; and Surgery.

Second---That all the chairs be filled upon the recommendation of the Medical Faculty, subject to the approval of the Board of Regents.

Third---That the appointment of all demonstrators and clinical assistants be left entirely with the Dental Faculty.

The Committee also recommended the following appointments, as suggested by the Medical Faculty, viz.: S. W. Dennis, M. D., D. D. S., Prof. of Principles and Practice of Operative Dentistry; A. F. McLain, M. D., D. D. S., Prof. of Dental Pathology and Therapeutics; C. L. Goddard, A. M., D. D. S., Prof. of Dental Art and Mechanism; M. W. Fish, M. D., Prof. of Physiology; A. W. Perry, M. D., Prof. of Chemistry; Wm. Leavitt, M. D., Prof. of Anatomy; and W. E. Taylor, M. D., Prof. of Surgery.

Thus it will be seen, the organization is complete. The positions for the most part are filled by men of recognized ability and standing in the profession, and all are doubtless well qualified for the work they have undertaken.

This institution, backed by the University, will, without doubt, succeed, and effect a great work for the dental profession, (and the public as well) of the Pacific Coast. It is eminently important that it should establish a high standard of qualifications and attainments; there is every incentive to this course. The great want of the profession is a higher culture and education, and to whom are we to look for this, if not to our colleges, and especially to those that are now organizing, and those that may in the future be established. The profession will expect good and efficient work from the California Dental College.

